# Enabling research capacity strengthening within a consortium context: a qualitative study

**DOI:** 10.1136/bmjgh-2022-008763

**Published:** 2022-06-28

**Authors:** Abiola Aiyenigba, Pierre Abomo, Neele Wiltgen Georgi, Imelda Bates, Justin Pulford

**Affiliations:** Department of International Public Health, Liverpool School of Tropical Medicine, Liverpool, UK

**Keywords:** qualitative study, health systems

## Abstract

**Introduction:**

We explore how health research consortia may be better structured to support research capacity strengthening (RCS) outcomes. The primary research questions include: in what ways do consortium members perceive that they and their respective institutions’ research capacity is strengthened from said membership? And, drawing on member experiences, what are the common factors that enable these perceived gains in research capacity to be realised?

**Methods:**

A qualitative study set within the ‘Developing Excellence in Leadership, Training and Science’ (DELTAS) Africa initiative. Semi-structured interviews were completed with 69 participants from seven institutions across six African countries belonging to three DELTAS Africa consortia. Data were analysed thematically via a general inductive approach.

**Results:**

A diverse array of perceived individual and institutional benefits of RCS consortium membership were reported. Individual benefits included access to training, resources and expertise as well as research and research leadership opportunities. Many institutional-level benefits of consortium membership were also driven through investment in individuals. Four enabling factors presented as especially influential in realising these benefits or realising them to a greater extent. These included: (1) access to funding; (2) inclusive and engaging leadership; (3) a diverse array of facilitated interactions for consortium members; and (4) an efficient interface between a consortium and their respective member institutions.

**Conclusion:**

Many reported benefits of RCS consortium membership were realised through funding access, yet attention to the other three enabling factors may further amplify the advantages conferred by funding access or, when funds are insufficient, ensure worthwhile gains in RCS are still achieved.

**WHAT IS ALREADY KNOWN ON THIS TOPIC**

- The use of research consortia is an increasingly common approach to strengthen the health research capacity in low-income and middle-income countries.
- However, there is very little evidence to inform how consortia may be structured to optimise research capacity strengthening (RCS) outcomes.

**WHAT THIS STUDY ADDS**

- Four enabling factors presented as especially influential to optimising RCS outcomes within a consortium context.
- These included: (1) access to funding; (2) inclusive and engaging leadership; (3) a diverse array of facilitated interactions for consortium members; and (4) an efficient interface between a consortium and their respective member institutions.

**HOW THIS STUDY MIGHT AFFECT RESEARCH, PRACTICE AND/OR POLICY**

- Many of the reported benefits of RCS consortium membership were realised through funding access, yet attention to the other three enabling factors may further amplify the advantages conferred by funding access or, when funds are insufficient, ensure worthwhile gains in RCS may still be achieved in a consortium context.

## Introduction

The ability to produce robust, locally appropriate research is an essential component of an effective health system.^1 2^ Unfortunately, there exist large global inequalities in the capacity to undertake health research. For example, low-income countries received only 0.2% of 69 420 biomedical grants listed in the World RePORT platform for the year 2016^3^ and countries belonging to the WHO African Region produced only 1.3% of global health research publications in 2014.^4^ One means by which health research capacity can be strengthened is through the formation of research capacity strengthening (RCS) consortia. Consortia bring together individuals and organisations with varying levels of expertise, experience and resources to work towards a common goal.^5^ In an RCS-focused consortium, the common goal is enhanced research capacity for some or all its members. This may be achieved through such things as skills transfer, shared learning or access to resources made available to consortium members.^6 7^ Research capacity strengthening consortia in a global health context often consist of members from both high and lower-middle income countries with the former most often cast in the lead role,^8^ although so-called ‘Southern-led’ consortia consisting exclusively or predominantly of low-to-middle-income country members are increasingly common.^9 10^

Evidence for the effectiveness of RCS interventions of any form, including consortium-based initiatives, is poorly developed.^11 12^ Without such evidence, it is not possible to reliably assess the full potential of consortia to facilitate RCS either independently or relative to other forms of RCS (eg, academic scholarships, infrastructure grants or mentoring schemes). In lieu of robust, long-term outcome evaluation, several examples of health RCS consortia have been presented in the published literature, many of which retrospectively report on programme achievements and/or challenges and lessons learnt in pursuit of such achievements.^10 13–15^ These accounts provide useful insights into the process and performance of consortia-based approaches to health RCS. Nevertheless, many such accounts lack scientific independence or research rigour and very few examine the relationship between process and performance in-depth or through a critical lens. A recent review of management processes within health RCS consortia highlights many of these limitations^16^: 37 of the 55 publications included in the review were commentaries; 10 of the 18 original research publications were authored by members of the respective consortia; discordant perspectives were present in only one publication (all others were reports of successful consortia); and relatively few extended beyond descriptive accounts of management activities and/or RCS outcomes to more detailed analyses of the linkages between the two.

To partly address these shortcomings, we present findings from a prospective, scientifically independent qualitative study set within the context of a Southern-led, consortia-based health RCS initiative. The study draws on a diverse array of participants from multiple RCS consortia and examines the experience of consortium membership from both an individual and institutional perspective. The primary research questions include: in what ways do consortium members perceive that they and their respective institutions’ research capacity is strengthened from said membership? And, drawing on member experiences, what are the common factors that enable these perceived gains in research capacity to be realised (or realised to a greater extent)? It was anticipated that the study findings would strengthen the evidence base pertaining to RCS; in particular, through advancing current understanding as to how consortia may be structured to better support RCS outcomes.

## Methods

We conducted a qualitative study using semi-structured interviews to examine the experience of consortium membership from the perspective of both participating individuals and institutions with the aim of drawing out those factors that enable RCS within the consortium context.

### Study setting

The study was set within phase one (2016–2021) of the ‘Developing Excellence in Leadership, Training and Science’ (DELTAS) Africa initiative. DELTAS Africa phase one was a US$100 million RCS programme implemented by the African Academy of Sciences’ Alliance for Accelerating Excellence in Science in Africa (AESA) with the objectives to: (1) develop world class researchers and research leaders to address African health and research priorities; (2) support the training and development of careers in scientific research; (3) nurture mentorship, leadership and equitable collaboration in science, as well as engage with public and policy stakeholders; and (4) cultivate professional environments to manage and support scientific research. DELTAS Africa phase one grants were awarded to 11 African-led RCS consortia collectively spanning 54 institutions from across the continent. This study draws on data collected from 3 out of these 11 consortia.

Consortium A (CA) consisted of four research institutions, and eight universities from six African countries, in collaboration with seven universities from the global North. The secretariat was situated at the lead institution in East Africa and consisted of 10 staff members who managed consortium activities. CA supported up to 200 PhD and postdoctoral fellows, recruited across multiple cohorts, registered across the eight African partner universities in East, West and Southern Africa. The consortium was formed in 2008 and DELTAS Africa was one of five funding initiatives supporting CA at the time of study. All institutions belonging to the consortium were officially English-speaking.

Consortium B (CB) consisted of one research institution and four universities based in West (4) and Central (1) Africa and three Northern collaborating partners. Three of the five African institutions were English-speaking and two French-speaking. The secretariat was located within the lead institute in a French-speaking West African country where consortium administrative and training activities at partner institutions were overseen by a staff of three. CB supported 15 PhD and postdoctoral fellows collectively registered across the five sub-Saharan Africa member institutions. CB was formed in 2015 building on a pre-existing consortium with similar research themes.

Consortium C (CC) consisted of three research institutions and four universities located across six countries in East and West Africa. Four of the consortium member institutions were English-speaking and three French-speaking, with the secretariat located in the lead institution in a French-speaking West African country. CC supported approximately 40 Masters, PhD and postdoctoral fellows registered at 16 universities and research institutions. CC started in 2016 with the financial support of the DELTAS Africa initiative, building on an earlier collaboration which commenced in 2009 with similar research themes. Consortium activities were coordinated by eight administrative staff members based at the secretariat.

### Consortium and participant selection

Consortia were purposively selected to reflect a balance in geographical location across sub-Saharan Africa, inclusive of Anglophone and Francophone nations. Following consortia selection, and subsequent approval from the respective consortia directors (see ‘procedures’ below), purposive sampling was further employed to select member institutions belonging to each consortium (ie, data were only collected from a subsample of consortia member institutions as opposed to all member institutions) and to recruit participants from each selected member institution. The lead institution of each consortium, or the institution hosting the secretariat, was selected in each case along with one-to-two member institutions. The latter were selected based on reported variation in existing research capacity and/or level of engagement in the respective consortium. Participant selection was carried out in consultation with programme managers from each consortium and, where required, from a consortium-nominated contact person within each participating member institution. The programme managers/contact persons assisted in identifying consortium members at various career stages and in various occupations to provide wide-ranging perspectives of consortium experiences. Participants were recruited until data saturation was reached in the study focal areas (see ‘data analysis’ below).

### Procedures

Data collection took place in six African countries (three Anglophone and three Francophone), across seven institutions from February to August 2019. Approval was first sought from the director of each of the three selected consortia. This was facilitated by the provision of an information sheet describing the study and by a support letter provided by AESA indicating their support, although equally noting that participation was voluntary and at the discretion of each consortium director. Once approval was obtained from consortium directors, dates for site visits and interview schedules were organised in consultation with the respective programme managers/contact persons. Prior to any site visit, the study information sheet, and an invitation to participate was sent via email to all potential participants.

Interviews took place in-person at designated office spaces at participating institutions. Prior to the interview, participants were given an information sheet describing the purpose of the research, what was expected from them, the duration of the interview, expected risks and benefits, that participation was confidential and voluntary and that they can withdraw at any time with no negative repercussions. There were no refusals or drop-outs.

Interviews were conducted using semi-structured interview guides developed by the research team and informed by the study objectives. In most cases, interviews were conducted in the participants primary language (either French or English). In a small number of cases Francophone participants were interviewed in English, although all were fluent English speakers. All interviews were conducted by two research team members one of whom was fluent in both English and French, the other fluent in English only. All interviews were audio recorded and lasted approximately 60 min on average. The researchers had email access to participants in the event clarifications were required after data collection. All interviews were transcribed. French-language transcriptions were professionally translated into English language before analysis.

The research team were scientifically independent of the three selected consortia, although they belonged to a larger research programme designed to support learning across the DELTAS Africa network. As such, the research team were sympathetic to, and supportive of, DELTAS Africa objectives and were broadly considered members of the DELTAS Africa network. Nevertheless, the research team were not accountable for, responsible for reporting on or in any way impacted by, consortia performance nor were consortia in any way accountable to the research team. Participants were aware of the research team’s role within DELTAS Africa and, as such, would likely have afforded them some form of ‘insider’ status. Insider status may have been further reinforced by the fact that all interviews were conducted by PhD graduates of African descent with lived experience of attending higher education institutions in either Anglophone or Francophone Africa.

### Data analysis

Data analysis was informed by a general inductive approach,^17^ aligning emerging themes identified in the data with predetermined focal areas relevant to the overarching study objectives. Interview transcripts were initially coded by the lead author (AA), resulting in a data framework and draft narrative presenting emerging themes and subthemes under constructs of ‘Perceived RCS benefits of consortium membership, individual and institutional’, ‘Perceived challenges of consortium membership, individual and institutional’ and ‘Common enablers of RCS’. The framework and draft narrative were then shared with two coauthors (NWG and JP) for critical review and collectively revised over several iterations. Final coding decisions were agreed by consensus opinion. NVivo software (V.12) was used to support the data analysis.

### Patient and public involvement

No patients or members of the public were involved in the design, conduct or reporting of this study.

## Results

### Participant characteristics

Semi-structured interviews were completed with a total of 69 participants from seven institutions across the three consortia. Participants consisted of consortia funded masters, doctoral and postdoctoral fellows, academic faculty attached to the consortia, as well as consortium management, administrative and support staff. The academic faculty included academic supervisors, lecturers, departmental professors and heads of department who had a role within the consortium, although were primarily employees of the respective member institutions. Participants were regionally located in East, West and Central Africa. Participant characteristics and distribution are shown in table 1.

**Table 1:** Participant characteristics

Participant characteristics		CA	CB	CC	Total
		n	n	n	N (%)
Total no. of participants		27	16	26	69 (100)
Gender	Male	15	10	17	42 (61)
	Female	12	6	9	27 (39)
Position at consortium	MSc fellow	0	0	4	4 (6)
	PhD fellow	10	2	10	22 (32)
	Postdoctoral fellow	2	2	1	5 (7)
	Academic faculty	5	4	4	13 (19)
	Management, administration and support	9	8	8	25 (36)
Duration of involvement with consortium activities in years	1	6	1	6	13 (19)
	2	5	3	10	18 (26)
	3	4	9	8	21 (30)
	4 or more	12	3	2	17 (25)
Geographical location of host institution	West Africa	0	12	19	31 (45)
	East Africa	27	0	7	34 (49)
	Central Africa	0	4	0	4 (6)
Primary language	English	27	3	8	38 (55)
	French	0	13	18	31 (45)

CA, Consortium A; CB, Consortium B; CC, Consortium C.

### Reported RCS benefits of consortium membership

Table 2 summarises the reported RCS benefits of consortium membership at both individual and institutional levels. As shown, reported benefits were many and varied and extended beyond established ‘staples’ of RCS consortia participation such as access to research funding, the provision of quality training and infrastructure development. A comparable range of benefits were generally reported across all three consortia, although not all were experienced to the same extent. Similarly, not all consortia members at either an individual or institutional level benefitted equally even within the same consortium. All three consortia were primarily geared towards supporting the training and development of early career researchers and, as such, it was the various fellows who seemingly benefitted most. Lead institutions also often seemed to benefit more than member institutions through a greater concentration of consortium resources. Even if benefit allocation remained unequal, common enabling factors that supported the realisation, or realisation to a greater extent, of reported RCS benefits associated with all forms of consortium membership were apparent across the three cases.

**Table 2:** Reported RCS benefits of consortium membership at individual and institutional levels

Reported RCS benefits	
Individual	Institutional
Access to specialist training—hard and soft skill development. Access to funding to undertake and lead own research projects. Access to consortia resources (across partner institutions) including specialised equipment. Access to consortia networks. Access to career supportive policies and practices via consortia (eg, provision of childcare support). Access/exposure to senior academic expertise within consortia. Access/exposure to key research end-users, including Government officials. Enhanced supervision through access to a stronger supervisory ‘pool’ and through more robust supervisory practices. Greater opportunities for broader research participation (eg, contributing to consortia research initiatives in addition to primary research). Greater opportunities for research grants, research publications and conference/meeting attendance. Greater opportunities for supervisory/teaching/leadership roles. Reputational enhancement through training received, association with consortia and through exposure to new networks/influential stakeholders.	Investment in infrastructure development including upgrading of facilities and procurement of specialist equipment. Access to funding and consortia resources including staff and training. Enhanced networking and research collaborations. Adoption of consortia-initiated ‘good practices’ and policies (eg, adoption of financial reporting templates or supportive childcare policies). Enhanced reputation, through consortia membership and associated research impact. Better capacitated workforce. Expanded workforce—in administrative, professional, teaching and research roles. Career development opportunities for existing staff (eg, PhD fellows recruited from existing staff).

RCS, research capacity strengthening.

### Enablers of RCS within the consortium context

Analysis within this section draws on both the description of factors that were considered direct enablers within the study data as well as experienced barriers to programme implementation or (desired) programme impact. The latter contribute to the analysis of enabling factors supporting RCS in the sense that they allow us to consider what factors might need to be overcome (or mitigated) to optimise consortia RCS potential. Our analysis revealed four overarching themes that may be considered central to optimising RCS in a consortium context: (1) funding, (2) leadership, (3) interaction and (4) interface. Each theme is discussed in turn.

#### Funding

… in my own field from lab techniques there is a big gap between us and [non-consortium] fellows at the university. Here [within the consortium] we have the opportunity to collect data easily, on time. We have the opportunity and the material to conduct our research in a lab at any moment, but at the university this is not the case. Some of our colleagues there can spend 3–4 years without nothing. They just register every year, but there is no fund and material in the lab to work. PhD fellow, CC

Funding presented as a key enabling factor to both individual-RCS and institutional-RCS in all three consortia. The monies received through the DELTAS Africa initiative supported: fellows to lead and/or engage well-resourced research projects (‘learn by doing’); access to high-quality, multidimensional training for members and (in some cases) broader institutional research and research support staff; investments in infrastructure development; employment and/or stipend for fellows and (in some cases) research support staff within home institutions; and supported networking, dissemination and community and public engagement. In short, many of the reported and apparent RCS benefits of consortium membership, from either an individual-perspective or institutional-perspective, would not have been realised (or realised to the same extent and/or within the same time frames) without access to the financial resourcing that consortium membership conferred.

Conversely, funding represented a major barrier to sustaining and/or building on RCS achieved within consortium lifespans. Participant responses often suggested an over-reliance on obtaining continued DELTAS funding from future versions of the scheme for programme continuity as opposed to exploring alternative or more innovative funding mechanisms.

We depend on project funding. Our budget, I think, is 95% project funding, so we don’t have a lot of co-support. PhD Fellow, CB

Available funding could also create inequities within member institutions between those colleagues who belonged to a consortium and those who did not. These inequities manifested in terms of both access to resources, for such things as training participation, research support and career supportive practices such as childcare provision to attend conferences, as well as distortions in academic salary scales. For example:

He’s [a consortium funded postdoctoral fellow] supposed to receive 1.5 million [in salary support]. At the university, this is not possible because the professor himself will not receive 1 million, why his postdoc will receive? We have this kind of conflict that it blocked the progress in implementing a project like [consortium name]. Management, CC

#### Leadership

I really liked the presence of [name of consortium director]. I understand that he is close to young people. He doesn’t only give the subject, he is there. I really felt that. The fact that he came really touched me. I tell myself that [consortium name] is a bit like senior, adult and youth. I liked that, this link between him and the beneficiaries. PhD fellow, CC

The leadership style of consortia leaders could be highly impactful on the respective members at an individual level. This was especially true for the many early career researchers who relished opportunities within the consortium to meaningfully engage with directors and senior scientists pre-eminent in their respective fields. As illustrated by the preceding quote, these interactions were especially powerful when consortium leadership were willing and able to engage with junior members on a more personal, egalitarian level. Fellows often reported adapting their own approaches to teaching, supervision or professional interaction based on their own positive experiences engaging with consortium leadership. For example, postdoctoral research and PhD fellows at both CB and CC described modelling their supervision practices with junior peers on the same approach and style their consortium-appointed supervisors employed with them.

Performance and ‘high functionality’ in critical implementation roles such as project management, were also highly valued. In CB, for example, the project manager at the time of interview was considered responsible for improving the efficiency of consortium activities as compared with his predecessors:

We had lost actually two good other persons [in project management roles] when we started off because they couldn’t be patient, they wanted to move on to other things and so they left. So we lost, but it was a good thing they left because this good guy came. Yeah, he’s been really, really phenomenally helpful in pushing through this…so when I said things have been reduced from 6 and 8 weeks, to 2 weeks then it is due to him. Management, CB

Leadership structures and practices were also central to facilitating institutional-level RCS. The more engaged leaders of member institutions were in consortium oversight and decision-making structures, the greater the apparent impact of institutional RCS as well as the potential for sustained change over time. CA had more formal processes to facilitate this as compared with CB and CC, including an annual forum for Vice-Chancellors from all member institutions to ‘sustain institutional buy-in and support institutionalisation efforts’ (Extract from terms of contract, CA). The value of engaged leadership at the member institution level was often most apparent when such leadership changed, occasionally resulting in disrupted ‘consortium-member institution’ arrangements which would take time to resolve:

The communication between [consortium name] and the school here, it has been a bit difficult because there’s been too much change in the focal point here. First, it was [name of institutional lead] and then [name of replacement lead]. It destabilized a bit the relationship that they had. They lost their strategic place within the consortium related, for example, to finance management. They lost some access to some equipment like meeting rooms. It has impacted a bit how they train. Faculty, CC

#### Interaction

That’s why I’m talking about exchanges. As Montaigne said: “You must rub your brain against that of others.” It’s always good to know what others are doing, to see improvement, to have a better perception of what you’re doing and what you need to do. Support staff, CB

In addition to funding and leadership, ‘interaction’ emerged as a third key RCS enabler. Interaction was a multidimensional theme, inclusive of supportive interactions across: the academic hierarchy in sub-Saharan Africa; institutions belonging to the same consortium; consortium and non-consortium staff within the same institutions; sub-Saharan Africa regions, including both Anglophone and Francophone; Southern and Northern researchers and research institutions; academic disciplines and research and research support services; and across sectors. While funding was essential to enabling many (but not all) of these interactions, each consortium—and the broader DELTAS Africa initiative—was also deliberately constructed to facilitate interaction opportunities. For example, CC appointed a full-time policy facilitator tasked with ‘working at the interface of our research and policy stakeholder community, to appraise and exploit opportunities for policy engagement and impact and to provide support as to how best scientists can link with policy’ (extract from the consortium’s theory of change).

Interaction as an enabling factor was readily and frequently apparent within the context of fellows discussing the experience of consortium membership on their individual development.

The big impact I would like to highlight here is the connection. That is something really nice that I like being in that program because that program allows me to be in touch to meet some other African colleagues during that training, during that conference I attended and so on. Postdoctoral fellow, CC

However, as the excerpt below demonstrates, interaction was considered an RCS enabler at the institutional level as well.

And I see that the more you practice it, the more you will become the best at it and the more you will help other people to become better. Especially the Masters students [enrolled at home institution], we worked with them a lot and they’ve appreciated. PhD fellow, CA

While interaction was a multidimensional theme, one-specific type of interaction—the relationship between consortium fellows and their appointed supervisors—stood out as especially influential. Supervisors included senior and/or experienced academics appointed from both the fellows’ home institution and from within the wider consortium. Thus, the ‘pool’ (and, arguably, quality) of potential supervisors available to fellows was expanded due to consortium membership. Institutions also benefitted through the specialist training provided by consortia to institutionally appointed supervisors or through the broader ‘adoption’ of supervision policies introduced within the context of consortia participation. CA partner institutions, who were particularly focused on the institutionalisation of RCS gains, demonstrated this by implementing supervision policies adopted from the consortium including supervision contracts which specified roles and expectations of both research supervisors and their students.

Supervision, while an enabling factor when operating well, presented as a barrier when it did not. Consortium supervisors often had limited influence on host institution supervisors’ behaviour and supervisors at home institutions reported having insufficient time to support fellows due to existing teaching and research commitments. In a further reflection of the inequities that generous consortium funding could create, some home institution-appointed supervisors also felt that the financial support that trainee fellows benefited from was not extended to them despite the central role they played in successful completion of research projects and by extension, consortium activities. This perceived financial inequity became a disincentive to full commitment by supervisors in some cases.

#### Interface

So there’s a lot of lobbying that has to take place [between the consortium secretariat and member institutions], a lot of negotiations, a lot of diplomacy in your communication. You don’t just say, I want this report at this time. No. You might not get it. Support Staff, CA

The interface between consortia and member institutions emerged as a prominent, sensitive and often highly problematic form of interaction warranting careful consideration. Both the consortia and the respective member institutions had their own practices, policies and internal bureaucracies that could be complex and, at times, discordant with each other. Consortia management practices, as described elsewhere,^18^ varied widely yet were heavily influenced by funding requirements and were generally consortium-specific constructs independent of, or only partially embedded in, host institutions. Consortia research support staff noted that challenges related to the consortium-member institution interface were often exacerbated when their respective fellows did not promptly report problems (either personal or professional). Consortia fellows, on the other hand, reported uncertainty regarding ‘who does what’ resulting from a lack of understanding of the dynamics and nuances between consortium and institutional administrative processes. Challenges could be further complicated when the consortium-member institution interface crossed an Anglophone–Francophone divide due to both language constraints and differing organisational structures. For example, postgraduate training and pathways to progression had to be modified at CB and CC Francophone partner institutions to fit in with anglophone-style indicators. Similarly, member institutions geographically distant from lead institutions often experienced more difficulties aligning with consortium standards as they had less access to consortia support staff who, in turn, were typically less familiar with member institution bureaucracies as compared with lead institutions where they were mostly based.

Consortium experience, staffing structures and governance models, as well as effective communication practices, served to mitigate challenges that arose from the consortium-member institution interface. All three case consortia expanded on pre-existing programmes established prior to the DELTAS Africa initiative. The continuity in structure and staffing and (perhaps most importantly) the accrued experience all served to strengthen consortium understanding of, and ability to navigate, the bureaucracies and contextual constraints of member institutions. In addition to staff retention, other staffing structures within each consortium were also influential. Embedding consortium-funded staff within member institutions could serve to improve the consortium-member institution interface through the greater understanding and influence that this enabled. Embedded staff were also well placed to facilitate institutional RCS. For example, embedded postdoctoral ‘training’ fellows at CC partner institutions became a valuable institutional resource through their conduct of regular needs assessments to identify and coordinate training and support activities for CC fellows, CC research support staff and, by extension, to non-consortium staff and students who could access and participate in available training.

There have been trainings that have been organized by [consortium name], but which have not only targeted [consortium name] students. There were really many of our students and even in other services that are not related to the students who were recruited, who were able to benefit from this capacity building. Faculty, CB

The consortium-member institution interface was also influenced through recruitment strategies. In CA, for example, consortium fellows were recruited from the core faculty of member institutions yet retained their institutional positions. These fellows were not only able to lend their experience and influence to support consortium activities, but they were also well-positioned to serve as ‘agents of change’ within their home institutions and were less prone to being lost to the institute (along with their newly gained research and research leadership skills and expertise) at the conclusion of the DELTAS programme. In a similar vein, when leadership from member institutions were represented within consortium governance structures then this also provided an opportunity for bi-directional influence for mutual benefit (as discussed in ‘leadership’ above).

## Discussion

Our study findings revealed that both individuals and their respective research institutions can potentially realise a diverse array of research capacity gains through consortium membership. Individual benefits were primarily related to greater access to training, resources and expertise as well as greater opportunities to engage in essential research and research leadership activities. Many (but by no means all) of the reported institutional-level benefits of RCS consortium membership such as a better capacitated and/or expanded workforce were also driven through investment in individuals. This finding highlights the potential inter-relationship between individual-level and institutional-level RCS, although deliberate strategies were employed to facilitate these kinds of ‘dual’ benefit. For example, recruiting PhD or postdoctoral fellows from existing faculty staff of consortium member institutes or requiring consortium fellows to teach and take on supervisory responsibilities at their home institute.

Four common enabling factors were identified that served to maximise the benefits of RCS consortium membership and/or mitigate the challenges faced. These included funding, leadership, interactions and interface. The access to funding that consortium membership conferred was essential to the realisation of many perceived benefits such as undertaking specialist training, infrastructure development and, indeed, allowing often complex (and expensive) research projects to be implemented in good time and in full. ‘Generous funding’ has been reported as an important factor in institutionalising RCS^19^ and Southern research organisations have previously expressed a preference to collaborate with Northern partners as opposed to Southern counterparts on the basis that this is more likely to result in greater funding access.^20^ Thus, our findings further underline the necessity of funding access to both the research and RCS endeavours in the global South. Yet they also demonstrate the potential for Southern-led RCS consortia to be effective ‘mechanisms’ for funding access even if, as was the case with DELTAS Africa, the funding origin was primarily from Northern sources. This is further indicated by the fact that many, if not all, of the perceived benefits of consortium membership reported in our study echo those reported for Northern-led RCS consortia.^14 21^ The reliance on consortium funding to maintain an expanded workforce or continue consortium-led initiatives presented a ubiquitous threat to long-term sustainability. This conundrum is unlikely to be resolved without meaningful growth in research and development investment among African nation states.^22^ However, RCS consortia can adopt strategies to reduce reliance on external funding over the longer-term or mitigate the consequences of losing financial support at the end of a funding cycle. Examples from this study include recruiting existing academic staff employed on secure long-term contracts into PhD or postdoctoral positions to ensure they were not lost to the member institute at the conclusion of their training and strengthening the capacity of member institutes to support consortium activities as opposed to establishing parallel consortium-specific support teams. Supporting member institutes to develop strong research offices, optimise income generation, advocate for national research funding and to promote accessible, alternative career pathways for consortium fellows also present as potentially effective mitigation strategies.

Not all benefits of consortium membership were dependent on funding access and where funding was essential, the resulting benefits in terms of individual-level or institutional-level capacity strengthening were further amplified through effective leadership, interactions or interface. For example, member institutions with representation in consortium governance were well placed to optimise mutually beneficial outcomes; and individual consortium members were readily able to transfer newly acquired knowledge and skills to non-consortium members when afforded teaching and supervisory responsibilities within their home institution. These findings highlight the potential for well-resourced, well-structured and well-led RCS consortia to be greater than the sum of their parts. Equally, the findings suggest that a well-led, well-structured consortium may still confer significant RCS opportunities even if funding is less than desirable: facilitating interactions between diverse groupings, role-modelling effective, engaging scientific leadership and providing quality postgraduate supervision, all stood out as RCS enablers that could be delivered at relatively minimal cost. Two recent studies, both also grounded within DELTAS Africa, similarly concluded that meaningful interactions (inclusive of those between junior and senior researchers and in the context of supervision) were essential to research and research leadership development.^23 24^ As with our study, Burgess and Chataway,^23^ further reported that DELTAS fellows regularly shared their experiences and/or resources gained through DELTAS Africa membership with institutional colleagues or visiting researchers from more resource poor groups. Interactions within the scope of RCS consortia, therefore, should be intentionally constructed to support both inward and outward capacity strengthening, that is, to support the development of consortium members and to allow consortium members to transfer acquired capacities to non-members.

Our study findings suggest the importance of an effective consortium/member institution interface cannot be overstated, perhaps especially so when member institutions are from multiple countries and linguistic regions. An effective interface improves navigation across often complex bureaucracies, making it easier for the consortium to implement activities and for individual and institutional members to benefit from these activities. Additionally, an effective interface ensures that consortium activities themselves are optimised to the respective setting. Our findings further suggest that the consortium/member institution interface improves over time as partners become increasingly familiar with each other’s bureaucracies and ways of working. This finding speaks to the efficiencies that can be gained from longer-term partnership, which is itself a recommended principle of effective RCS.^25^ Our study also found that embedding consortium support staff within member institutions may enhance the interface between the two. This finding is consistent with lessons drawn from North–South RCS consortia where the placement of consortium-appointed staff within Southern institutions has previously been recommended.^14 15^ Embedding or appointing consortium focal points in member institutions may be especially powerful for new partnerships, to help address potential friction at the interface between the two as the partnership is established.

Our study was not without limitation. Only 3 out of the 11 phase one DELTAS Africa consortia were included in the study and in the 3 consortia that we did include, not all individuals or member institutes were involved. Consortium experiences may have been different for members and member institutes who did not participate and, as such, the reported findings should not be considered representative of the entire DELTAS Africa initiative. Having said that, we optimised diversity within our cases to increase the generalisability of our findings and many of the perceived benefits and challenges and all four of the identified enabling factors were common across all three case consortia suggesting some degree of generalisability. As study data were collected during a single time point in year 4 of the 5/6 year DELTAS Africa phase one awards, it is also not possible to infer the sustainability or longer-term outcomes of perceived RCS benefits reported. In addition, our study design did not allow us to reliably assess the relative frequency or comparative impact of the various benefits of RCS consortium-membership conferred. Issues of equity and reciprocity, such as whether all partners contributed and/or benefitted in equal measure or relative to their respective needs, were not examined in detail. Thus, while issues relating to equity were recognisable in our findings, we were not able to reliably comment on the extent of any inequities or the degree to which (if any) the Southern-led nature of the case consortia mitigated the experience of inequity often reported in the context of North–South RCS initiatives.^26^

## Conclusion

A wide range of perceived benefits to both individuals and institutions may be obtained through membership of an RCS consortium. Four enabling factors present as especially influential in realising these benefits or realising them to a greater extent. These include access to funding, inclusive and engaging leadership, a diverse array of facilitated interactions for consortium members and an efficient interface between the consortium and their respective member institutions. Many of the reported benefits of RCS consortium membership may only be realised through funding access, yet attention to the other three enabling factors may further amplify the advantages conferred by funding access or, when funds are insufficient, ensure worthwhile gains in RCS may still be achieved in a consortium context. The primary recommendation resulting from this study, therefore, is that when planning, implementing or funding a RCS-focused consortium then funding, leadership, interaction and interface dimensions should be carefully considered. Detailed and convincing plans for optimising each of these four dimensions should be present in the proposal stage and performance across each dimension monitored over the consortium life cycle. Future research could then focus on the relative contribution of each of these dimensions to consortium outcomes as well as further exploration of practical strategies for optimising each dimension across a range of implementation contexts.

## Data Availability

Data are available upon reasonable request. Transcriptions of interviews with consortia staff are available from the corresponding author on request. They have not been made available as a data set because they cannot be readily de-identified without compromising anonymity.
